# Supplementary material for: Early stage NSCLS patients’ prognostic prediction with multi-information using transformer and graph neural network model
Source: eLife. 2022 Oct 4;11:e80547. doi: 10.7554/eLife.80547 (PMC9531948; doi:10.7554/eLife.80547)
Supplement: Supplementary file 1. [file elife-80547-supp1.docx]

| **Feature** | **Content** | **Count, %** |
| --- | --- | --- |
| Ethnicity | Caucasian (%);  Asian (%);  Hispanic/Latino (%);  African-American (%);  Native Hawaiian/Pacific Islander (%) | 95 (74.8%);  20 (15.7%);  6 (4.7%);  5 (3.9%);  1 (0.8%); |
| Smoking status | Former (%);  Current (%);  Non-smoker (%) | 80 (63.0%);  29 (22.8%);  18 (14.2%) |

Supplementary File 1: Ethnicity and Smoking information table of the external dataset.
